# Prevalence and risk factors for hypertension and diabetes among those screened in a refugee settlement in Uganda

**DOI:** 10.1186/s13031-021-00388-z

**Published:** 2021-07-05

**Authors:** Rachel W. Kubiak, Elinor M. Sveum, Zikama Faustin, Timothy Muwonge, Hussain Abbas Zaidi, Andrew Kambugu, Simon Masereka, Julius Kasozi, Ingrid V. Bassett, Kelli N. O’Laughlin

**Affiliations:** 1 Department of Epidemiology, University of Washington, Seattle, WA USA; 2 Department of Emergency Medicine, University of Washington, Seattle, WA USA; 3 Bugema University, Kampala, Uganda; 4 Infectious Diseases Institute, Makerere Univesity, Kampala, Uganda; 5 Swarthmore College, Swarthmore, PA USA; 6 Medical Teams International, Mbarara, Uganda; 7 United Nations High Commissioner for Refugees, Kampala, Uganda; 8 Department of Medicine, Massachusetts General Hospital, Harvard Medical School, Boston, MA USA; 9 Department of Global Health, University of Washington, Seattle, WA USA

**Keywords:** Hypertension, Diabetes, Uganda, Refugee, Health screening

## Abstract

**Background:**

Diabetes and hypertension are increasingly prevalent in low and middle income countries, but they are not well documented in refugee settlements in these settings. We sought to estimate the prevalence and associated characteristics of diabetes and hypertension among adults presenting for clinic-based HIV testing in Nakivale Refugee Settlement in Uganda.

**Methods:**

HIV-negative adults presenting to outpatient clinics for HIV testing at three health centers in Nakivale Refugee Settlement were enrolled from January 2019 through January 2020. Multi-lingual research assistants administered questionnaires aloud to ascertain medical history and sociodemographic information. The research assistants used standardized procedures to measure participants’ blood pressure to detect hypertension (systolic blood pressure ≥ 140 mmHg or diastolic blood pressure ≥ 90 mmHg), and conduct a point-of-care blood glucose test for diabetes (random blood glucose ≥11.1 mmol/L with self-reported frequent urination or thirst, or fasting blood glucose ≥7.0 mmol/L regardless of symptoms), as per Uganda Ministry of Health guidelines. We used χ-square or Fisher’s exact test to test for differences in disease prevalence by refugee status and log-binomial or Poisson regression models to estimate associations of immigration status and country of origin, respectively, with hypertension and diabetes while controlling for age, sex, education level, and body mass index.

**Results:**

Among 2127 participants, 1379 (65%) were refugees or asylum seekers and 748 (35%) were Ugandan nationals. Overall, 32 participants met criteria for diabetes (1.5%, 95% CI 1.1–2.1%) and the period prevalence was 2.3% (95% CI 1.7–3.0). There were 1067 (50%, 95% CI 48.0–52.2%) who met the criteria for pre-hypertension and 189 (9%, 95% CI 7.7–10.1%) for hypertension. These proportions did not vary by immigration status or country of origin in univariate tests or multivariable regression models.

**Conclusions:**

Hypertension was common and diabetes was uncommon among those screened in a Ugandan refugee settlement. Routine blood pressure screening should be considered in this setting. Additional research could develop diabetes screening criteria to help identify at risk individuals in this limited resource setting.

## Introduction

Non-communicable diseases (NCDs) are the leading cause of mortality worldwide, with nearly three quarters of NCD-related deaths occurring in low- and middle-income countries (LMICs) [1]. In addition, many LMICs are experiencing a double burden of disease with high prevalence of both NCDs and infectious diseases that stretch the priorities and funding of limited health systems [2]. These challenges are compounded when considering refugee populations. Humanitarian crises can cause disruptions in previously available health services, further weaken fragile health systems, and divert resources away from chronic disease management [2]. As a result, research has shown an increase in NCD complications in conflict settings [2, 3]. As the prevalence of NCDs continues to rise and humanitarian crises persist, countries and organizations responding to humanitarian crises have an obligation to address long-term management of NCDs [4].

In the Middle Eastern region, gaps seen with NCD treatment in refugee settings tend to mirror overall challenges and weaknesses within national health systems [5]. Data on NCD care in refugee settlements in sub-Saharan Africa is much more sparse. A national prevalence survey in Uganda estimated 26.5% of adults had hypertension [6] and 1.4% had diabetes [7] and the majority were unaware of their underlying medical condition [6, 7], highlighting the importance of expanding access to screening for NCDs. Only 80% of Ugandan health facilities offered blood glucose testing and 34% offered diabetes management in 2013 [8]. Rural clinics in Uganda continue to face challenges in training health workers and providing continuous support for diabetes care [9]. Additionally, the Ugandan essential medicines list includes medications for hypertension and diabetes management [10, 11], but there is limited availability of essential medicines throughout Uganda and there are disparities related to less access at public hospitals compared to private for-profit hospitals [5]. In refugee settlements in Uganda, the burden of disease and degree to which there are unmet medical needs is unclear. Improved understanding could help identify unmet medical needs, inform public health policies and goals, and inform medical resource allocation for this vulnerable population.

To assess the NCD burden within Nakivale Refugee Settlement in southwestern Uganda, we leveraged existing infrastructure for patients presenting to health centers and screened them for hypertension and diabetes.

## Methods

### Setting

This research was conducted in Nakivale Refugee Settlement in southwestern Uganda. Over 100,000 refugees live in the settlement; the majority of refugees are from the Democratic Republic of the Congo (DRC), Rwanda, Somalia, and Burundi, and a small minority are from other nearby sub-Saharan African countries. This research was conducted at three health centers in the settlement, Nakivale Health Center, Kibengo Health Center and Juru Health Center. There is a fourth health center in the settlement but it was not an enrollment site given its remote location and difficult access compared to the other sites. Refugees and Ugandan nationals can access clinical services free of charge at health centers in Nakivale, including free prescription medications for diabetes, hypertension, and HIV when indicated.

### Study population and procedures

These NCD data were collected as a part of a larger study on linkage to HIV care in Nakivale Refugee Settlement (PI: O’Laughlin, K23MH108440). Prior to initiation of the NCD component of this work, our research team met with the local implementing partner leadership team to ensure there was sufficient capacity to accommodate people newly diagnosed with hypertension and diabetes. We worked with these partners to create a referral protocol that specified who to refer (e.g. based on classification of blood pressure or diabetes diagnostic criteria) and the expected timeline for medical follow-up. Multi-lingual research assistants were trained in blood pressure and glucose measurement techniques prior to study initiation. Adults presenting for HIV testing were recruited from the outpatient department waiting areas at Nakivale, Juru, and Kibengo Health Centers. Inclusion criteria were 1) 18 years of age or older, 2) willingness to participate in routine clinic-based HIV testing, 3) not previously diagnosed with HIV, 4) no prior participation in the study in the preceding 3 months, and 5) able to understand the consent process and study procedures in Kiswahili, Kinyarwanda, Runyankore, or English. After giving written consent, participants verbally completed a questionnaire which was read to them by a research assistant who directly entered information into an electronic database. Data collected included sociodemographic information, medical history, and ongoing diabetes and hypertension treatment. Research assistants then measured participants’ height, weight, blood glucose using the FreeStyle Optium Neo Blood Glucose and Ketone Monitoring System, and blood pressure prior to conducting HIV testing. For blood pressure measurements, participants were seated for 1 min before the measurement was taken using a stethoscope for auscultation and a Veridian Healthcare Pro Kit sphygmomanometer. For those with an elevated blood pressure, the measurement was repeated two additional times 5 min apart for each additional measurement according to Ministry of Health guidelines [12]. Research assistants then conducted point-of-care blood glucose and HIV testing. All participants included in these analyses were enrolled between January 16, 2019 through January 13, 2020.

### Definition of endpoints

Endpoints were established using Uganda Ministry of Health guidelines [12]. We defined diabetes as a random blood glucose (RBG) ≥11.1 mmol/L with self-reported frequent urination or thirst, or fasting blood glucose (FBG) ≥7.0 mmol/L regardless of symptoms [12]. We used the lowest systolic and diastolic blood pressures to ascertain hypertension and defined it as both a binary and categorical outcome (including pre-hypertension), according to local guidelines (Table 1) [12]. In contrast to World Health Organization guidelines, all measurements were taken on the same day. We used standard definitions of body mass index (BMI) < 18.5 kg/m^2^ for underweight, 18.5 ≤ BMI < 25.0 kg/m^2^ for normal weight, and BMI ≥25.0 kg/m^2^ for overweight/obese [13].

**Table 1:** Definitions of study outcomes

Outcome	Category	Definition
Diabetes	Present/Absent	RBG ≥11.1 mmol/L with self-reported frequent urination or thirst, or FBG ≥7.0 mmol/L regardless of symptoms
Hypertension	Present/Absent	SBP ≥140 or DBP ≥90 mmHg
Hypertension	Absent	SBP < 120 and DBP < 80 mmHg
	Pre-hypertension	SBP 120–139 or DBP 80–89 mmHg
	Stage 1	SBP 140–159 or DBP 90–99 mmHg
	Stage 2	SBP 160–180 or DBP 100–110 mmHg
	Stage 2, severe	SBP > 180 or DBP > 110 mmHg

*DBP* Diastolic blood pressure, *FPG* Fasting blood glucose, *RBG* Random blood glucose, *SBP* Systolic blood pressure

### Statistical analyses

We tested for differences in descriptive statistics and study outcomes by refugee status using χ-square or Fisher’s exact test for categorical variables and Student’s t-test for continuous variables. We used the Agresti-Coull method to calculate 95% confidence intervals (CI) for the prevalence of diabetes and hypertension [14]. We also estimated the period prevalence of diabetes by including participants who met the criteria for diabetes or reported a prior diabetes diagnosis in the numerator. We estimated the number needed to screen as 1/prevalence. We used log-binomial regression models or Poisson regression with robust standard errors if the model failed to converge [15, 16] to estimate the associations of immigration status and country of origin, respectively, with hypertension and diabetes while controlling for age, sex, education level, and BMI. We performed statistical analyses using SAS version 9.4 (Cary, NC).

## Results

### Study population

Of the 2137 participants enrolled since NCD testing was introduced, 2127 (99.5%) received blood glucose testing and blood pressure measurement. Among these, 1379 (65%) were refugees or asylum seekers and 748 (35%) were Ugandan nationals. Ugandan nationals were more likely to be female (60% vs 54%, *p* = 0.005) and older (32.8 ± 11.2 vs 31.1 ± 11.0 years, *p* < 0.001) compared to refugees and asylum seekers (Table 2). After Uganda, the most commonly reported countries of origin were the DRC (*n* = 481, 23%), Kenya (*n* = 462, 22%), and Burundi (*n* = 366, 17%). Somalia (*n* = 3, 0.1%), Sudan (*n* = 2, 0.1%), South Sudan (*n* = 1, 0.1%), and other (*n* = 22, 1%) were also reported.

**Table 2:** Study population characteristics for diabetes and hypertension by refugee status

	Refugee/Asylum Seeker *N* = 1379 N (%)	Ugandan National *N* = 748 N (%)	*p*-value
*Demographic and clinical characteristics*			
Female	737 (54)	447 (60)	0.005
Age (years)	31 ± 11	33 ± 11	< 0.001
Education			
Never attended school	318 (23)	139 (19)	0.009
Some primary	679 (49)	416 (56)	
Completed at least primary	385 (28)	192 (26)	
Body mass index (kg/m^2^)			
< 18.5	98 (7)	52 (7)	0.178
18.5–24.9	1020 (74)	526 (71)	
> 25.0	256 (19)	163 (22)	
HIV diagnosis	43 (3)	73 (10)	< 0.001
*Diabetes*			
Prior diabetes diagnosis	13 (0.9)	8 (1)	0.779
Frequent urination or thirst	326 (24)	184 (25)	0.621
Diabetes diagnosis^a^	17 (1)	15 (2)	0.162
*Hypertension*			
Prior hypertension diagnosis	84 (6)	28 (4)	0.021
Currently taking hypertension medication	25 (2)	11 (2)	0.559
Diastolic blood pressure (mmHg)	76 ± 10	74 ± 10	0.004
Systolic blood pressure (mmHg)	114 ± 13	113 ± 14	0.005
Pre-hypertension^b^	698 (51)	368 (49)	0.412
Hypertension^c^	127 (9)	60 (8)	

Chi-square or Fisher’s exact test for categorical variables and Student’s t-test for continuous variables

^a^Frequent urination or thirst and non-fasting blood glucose ≥11.1 mmol/L or fasting ≥7 mmol/L

^b^Diastolic blood pressure 80–89 or systolic blood pressure 120–139 mmHg

^c^Diastolic blood pressure ≥ 90 or systolic blood pressure ≥ 140 mmHg

### Hypertension

Overall, 1067 (50%, 95% CI 48.0–52.2%) of participants met criteria for pre-hypertension at the time of their clinic visit and 187 (9%, 95% CI 7.7–10.1%) met criteria for hypertension. The number needed to screen to identify one new instance of hypertension was 15.3 people and did not vary substantially by refugee status or country of origin. Among those with hypertension, 129 were stage 1, 48 were stage 2, and 9 were stage 2 severe. At the time of screening, 112 (5%) of participants reported a prior diagnosis of hypertension.

Among the 112 participants reporting previously diagnosed with hypertension, 48 (43%) were hypertensive at the time of screening reflecting uncontrolled hypertension and this did not differ by refugee status (Ugandan nationals 35/84, 42%; refugees and asylum seekers 13/28, 46%). Sustained anti-hypertensive treatment was uncommon with 31 (28%) reporting use of prescribed anti-hypertensive drugs in the past 2 weeks and 15 (13%) using traditional remedies. Use of a prescribed anti-hypertensive drug among those with a prior diagnosis did not vary by the presence of hypertension at the time of screening (*p* = 0.761).

### Diabetes

Overall, 32 participants met the criteria for diabetes (1.5%, 95% CI 1.1–2.1%). The number needed to screen to identify one new case of diabetes was 78.7 persons. The large majority (*n* = 27, 87%) did not report a prior diabetes diagnosis and this did not vary substantially by refugee status (Ugandan nationals *n* = 12/15, 80%; refugees and asylum seekers *n* = 15/17, 88%). There were no significant differences in previously reported or new diagnosis of diabetes by refugee status or country of origin (Table 3). Overweight and obesity were more common among participants with diabetes (*n* = 10, 32%) than those without diabetes (*n* = 409, 20%), though this was not statistically significant (*p* = 0.08).

**Table 3:** Diabetes and hypertension by country of origin

	Rwanda *N* = 462 N (%)	Uganda *N* = 790 N (%)	Congo (DRC) *N* = 480 N (%)	Burundi *N* = 363 N (%)	*p*-value
*Diabetes*					
Prior diabetes diagnosis	2 (0.4)	8 (1.0)	7 (1.5)	3 (0.8)	0.437
Currently uses diabetes medication	4 (0.9)	7 (0.9)	4 (0.8)	1 (0.3)	0.737
Frequent urination or thirst	93 (20.1)	200 (25.3)	130 (27.0)	75 (20.5)	0.024
Elevated blood glucose^a^	7 (1.5)	15 (1.9)	7 (1.5)	5 (1.4)	0.886
Diabetes diagnosis^b^	7 (1.5)	15 (1.9)	5 (1.0)	5 (1.4)	0.670
*Hypertension*					
Prior hypertension diagnosis	23 (5.0)	29 (3.7)	39 (8.1)	19 (5.2)	0.008
Currently uses hypertension medication	5 (1.1)	12 (1.5)	10 (2.1)	8 (2.2)	0.538
Pre-hypertension^c^	218 (47.2)	389 (48.9)	256 (53.2)	193 (52.7)	0.176
Hypertension^d^	41 (8.9)	63 (8.0)	49 (10.2)	33 (9.0)	
Number needed to screen	11.2	12.5	9.8	11.1	

Chi-square or Fisher’s exact test for categorical variables and Student’s t-test for continuous variables

^a^Fasting blood glucose ≥11.1 mmol/L or non-fasting ≥7 mmol/L

^b^Frequent urination or thirst and non-fasting blood glucose ≥11.1 mmol/L or fasting ≥7 mmol/L

^c^Diastolic blood pressure 80–99 or systolic blood pressure 120–139 mmHg

^d^Diastolic blood pressure ≥ 90 or systolic blood pressure ≥ 140 mmHg

The period prevalence was 2.3% (*n* = 48, 95% CI 1.7–3.0%). Among the 21 (1%) participants who reported a prior diagnosis of diabetes, 15 (71%) reported taking prescribed diabetes drugs within the prior 2 weeks, 10 (48%) reported ever visiting a traditional healer for diabetes and 9 (43%) reported current use of herbal or traditional remedies for diabetes. There were 5 individuals who reported a prior diabetes diagnosis and met the criteria for diabetes at the time of screening, all of whom reported recent diabetes prescription drug use. Elevated blood glucose was more common among Ugandan nationals (3/8, 38%) than refugees and asylum seekers (2/13, 15%), though this was not statistically significant (*p* = 0.248).

### Multi-morbidity

**Fig. 1:**
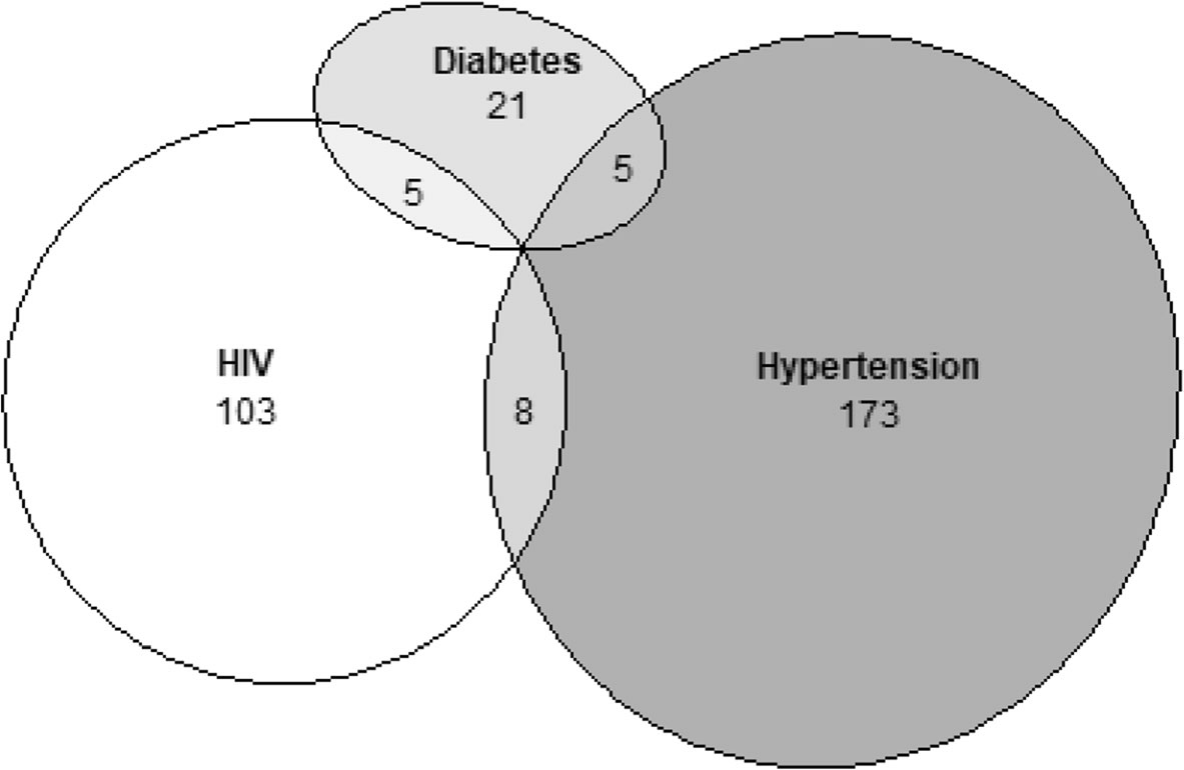
Venn diagram of the number of participants with diabetes, HIV, and hypertension

A total of 116 participants tested positive for HIV infection. Few participants had multi-morbidity (Fig. 1). In multivariable models, diabetes and hypertension were associated with age but not refugee status (Table 4) or country of origin (data not shown).

**Table 4:** Study population characteristics for diabetes and hypertension

	Diabetes		Hypertension	
	aRR (95% CI)	*p*-value	aRR (95% CI)	*p*-value
Age (per 5 years)	1.16 (1.00–1.34)	0.043	1.22 (1.16–1.28)	< 0.001
Sex				
Male	1	0.967	1	0.361
Female	1.02 (0.48–2.17)		0.87 (0.65–1.17)	
Education				
Never attended school	1	0.056	1	0.186
Some primary	3.30 (0.67–11.25)		0.80 (0.57–1.12)	
Completed at least primary	10.90 (0.94–129.52)		0.63 (0.32–1.25)	
Body mass index				
Low/Normal	1	0.085	1	< 0.001
Overweight/obese	1.97 (0.91–4.28)		2.66 (2.03–3.48)	
Immigration status				
Ugandan national	1		1	0.181
Refugee/asylum seeker	0.74 (0.36–1.49)		1.22 (0.91–1.62)	

*CI* Confidence interval, *aRR* Adjusted relative risk

## Discussion

Among 2127 adults presenting for routine HIV testing at health clinics in Nakivale Refugee Settlement, the prevalence of pre-hypertension and hypertension were high, while the prevalence of diabetes was low. The burden of hypertension and diabetes were similar across refugee status and country of origin. The majority of participants did not suffer from multi-morbidity.

Studies of NCD prevalence and interventions among refugee populations have overwhelmingly focused on the Middle East geographically [2, 4, 8, 10, 17]. There, health services are provided to those in refugee settlements through the existing urban health infrastructure of the host country. Our data showed that in Nakivale Refugee Settlement the opposite is happening; 35% of participants were Ugandan Nationals integrated into the refugee health system. Inadequate treatment for those with known conditions has also been observed previously [3, 17]. Among Syrian refugees in Jordan, hypertension and diabetes were the most prevalent NCDs and observed at similar frequencies as in the general Jordanian population [17, 18]. Similarly, we found no significant difference in burden of disease between refugee and Ugandan nationals. The period prevalence of 2.3% is higher than a 2014 national prevalence estimate of 1.4%, possibly due to higher diabetes prevalence in the countries of origin [7]. Interestingly, the prevalence of hypertension at these refugee clinics (8.8%) is considerably lower than the national prevalence estimate of 26.5% [6].

Blood pressure screening for pre-hypertension or hypertension in this population could be feasible and would have a high yield. A blood pressure test is non-invasive, low cost, and requires minimal training and infrastructure to administer. There is a strong association between hypertension and cardiac disease, as well as mortality [19]. Although technically the diagnosis of hypertension requires two or three high blood pressure measures at least one week apart, the ability to screen patients with a single visit in order to direct them to further care could have a large impact in diagnosing hypertension for earlier intervention and decreasing disease complications. Immigration status and country of origin were not significant predictors of hypertension indicating screening could be broadly implemented at all health centers in the refugee settlement. Outreach to better understand and address barriers to care faced by vulnerable and underserved populations, such as Somali nationals who are underrepresented in these data, could increase the impact of the program.

Increasing screening will inevitably place a greater burden on the health system to provide medications and clinic visits to more individuals. This will put additional stress on an already under-performing system [8, 9]. However, leveraging pre-existing HIV infrastructure as was done in this study already has been shown to be feasible and cost-effective when tailored to the appropriate population [20, 21]. Task shifting by using community health workers or peers can also be a cost-effective way to provide hypertension and diabetes care [22–24]. Community health workers can provide a variety of services including questionnaire-based screenings and referrals for testing, education around non-pharmaceutical disease management, and developing client self-efficacy for medication adherence and home-based disease management (e.g. glucose or blood pressure monitoring) [22–25]. Such a program would need to be well-managed, provide ongoing training and salary support for the community health workers, and be developed in the context of broader health systems strengthening programs [25, 26]. Additionally, Médecins Sans Frontières demonstrated the feasibility and effectiveness of providing hypertension and diabetes treatment for refugees and vulnerable host communities within a camp by conducting a clinical consult, lab draws, and drug delivery (including a three-month supply to stable patients) at the same visit [3].

Diabetes screening in this setting will need to be carefully considered given the large number needed to screen and the considerable resources required. Diabetes testing is complex, requiring a multi-step testing process and advanced laboratory capabilities. Additionally, established screening methods may not be as effective in this population. For example, thirst is a well-established symptom of diabetes, but may not perform well in this highly resource-constrained setting. Additional research is needed to identify sub-populations most at risk of diabetes and possibly develop modified screening guidelines in order to target diabetes testing for those most likely to benefit from treatment. Although not statistically significant in our data, there was a higher prevalence of diabetes among overweight or obese individuals, which could be an appropriate sub-population to screen.

Approximately three quarters of patients previously diagnosed with diabetes had access to prescription diabetes drugs and did not have elevated blood glucose at the time of their clinic visit, suggesting successful pharmacologic disease management for the majority of cases. Notably, all five previously diagnosed participants with continued elevated blood glucose at the time of their clinic visit also reported recent medication use, indicating a role for more intensive diabetes management in select cases. It may be that refugees with complex medical needs should be considered for more urgent resettlement so they can better care for their health needs.

Our study has several strengths. We are among the first to estimate the burden of hypertension and diabetes in a vulnerable refugee population in a settlement. We have a large sample size. We also have good ascertainment of hypertension, using three consecutive measurements.

Our study also has several limitations. We had a small number of diabetes cases, despite our large sample size, which likely limited our ability to detect an association between BMI and diabetes in multivariable analysis. Additionally, diabetes is difficult to test for and we could not confirm hyperglycemia with a repeat glucose test or follow-up HbA_1c_ testing, as recommended [12], so the true prevalence is likely lower than estimated. Conversely, relying on blood pressure measurements all taken on the same day, instead of the recommended 2 days, may overestimate hypertension prevalence [27]. Utilizing local criteria rather than the more stringent WHO criteria makes generalizing these findings to other contexts difficult. Furthermore, adults presenting for HIV testing at an outpatient clinic may not be a representative sample of the local population. Those presenting for care are either sicker or more health-conscious and therefore more or less likely to screen in for hypertension or diabetes than those not presenting to the health center. To the extent that HIV infection and antiretroviral therapies increase the risk of diabetes and that HIV is prevalent in the community, our estimate of diabetes, which excludes people with a prior HIV diagnosis, may underestimate the true overall prevalence [27–31]. Lastly, given stigma around HIV testing and the fact this study was nested in an HIV study, some of the population was not screened and hence underrepresented. For instance, there were only three Somalis involved in our study, while the population of Somalis in Nakivale was 13,397, demonstrating the lack of true representation for this specific group of refugees. Individual or focus group discussions with community leaders and members could help identify barriers to care in this setting as a first step towards designing targeted interventions.

## Conclusions

At health centers in Nakivale Refugee Settlement in Uganda attended by refugees and Ugandan nationals, elevated blood pressure was common and frequently unknown or uncontrolled. Testing could be incorporated into the clinic visit flow and, if sustained monitoring and treatment is provided, could improve long-term health outcomes. Diabetes prevalence was low. Given the challenges associated with diabetes screening and high frequency of severe outcomes associated with this disease, focused screening of higher risk individuals should be considered in this setting.

## Data Availability

The datasets used and/or analysed during the current study are available from the corresponding author on reasonable request.

## Abbreviations

BMI: Body mass index
CI: Confidence interval
DRC: Democratic Republic of the Congo
FBG: Fasting blood glucose
LMICs: Low- and middle-income countries
NCDs: Non-communicable diseases
RBG: Random Blood Glucose

## References

[1] Alawa J, Bollyky TJ (2020). A silent crisis: the rise of non-communicable diseases in refugee settings: council on foreign Relations.

[2] Aebischer Perone S, Martinez E, du Mortier S, Rossi R, Pahud M, Urbaniak V (2017). Non-communicable diseases in humanitarian settings: ten essential questions. Confl Heal.

[3] Kayali M, Moussally K, Lakis C, Abrash MA, Sawan C, Reid A (2019). Treating Syrian refugees with diabetes and hypertension in Shatila refugee camp, Lebanon: Médecins Sans Frontières model of care and treatment outcomes. Confl Heal.

[4] Ruby A, Knight A, Perel P, Blanchet K, Roberts B (2015). The effectiveness of interventions for non-communicable diseases in humanitarian crises: a systematic review. PLoS One.

[5] Armstrong-Hough M, Kishore SP, Byakika S, Mutungi G, Nunez-Smith M, Schwartz JI (2018). Disparities in availability of essential medicines to treat non-communicable diseases in Uganda: a Poisson analysis using the service availability and readiness assessment. PLoS One.

[6] Guwatudde D, Mutungi G, Wesonga R, Kajjura R, Kasule H, Muwonge J, et al. The epidemiology of hypertension in Uganda: Findings from the national non-communicable diseases risk factor survey. PLoS One. 2015;10(9):e0138991. 10.1371/journal.pone.0138991 PMC4583385 26406462

[7] Bahendeka S, Wesonga R, Mutungi G, Muwonge J, Neema S, Guwatudde D (2016). Prevalence and correlates of diabetes mellitus in Uganda: a population-based national survey. Tropical Med Int Health.

[8] Atun R, Davies JI, Gale EAM, Bärnighausen T, Beran D, Kengne AP (2017). Diabetes in sub-Saharan Africa: from clinical care to health policy. Lancet Diabetes Endocrinol.

[9] Birabwa C, Bwambale MF, Waiswa P, Mayega RW (2019). Quality and barriers of outpatient diabetes care in rural health facilities in Uganda - a mixed methods study. BMC Health Serv Res.

[10] Valluri S, Gaziano TA (2013). Progress in national and regional guidelines development and deployment for the clinical prevention and control of CVD and diabetes in Africa. Prog Cardiovasc Dis.

[11] Organization WH. Global Health Estimates 2016: Deaths by cause, age, sex by country and by region, 2000-2016. Geneva: World Health Organization; 2018.

[12] Uganda. MoH (2016). Uganda Clinical Guidelines 2016: National Guidelines for Management of Common Conditions.

[13] Weir CB, Jan A (2020). BMI Classification Percentile And Cut Off Points.

[14] Brown LD, Cai TT, Dasgupta A (2001). Interval estimation for a binomial proportion. Stat Sci.

[15] Zou G (2004). A modified poisson regression approach to prospective studies with binary data. Am J Epidemiol.

[16] Spiegelman D, Hertzmark E (2005). Easy SAS calculations for risk or prevalence ratios and differences. Am J Epidemiol.

[17] Ratnayake R, Rawashdeh F, AbuAlRub R, Al-Ali N, Fawad M, Bani Hani M (2020). Access to care and prevalence of hypertension and diabetes among Syrian refugees in northern Jordan. JAMA Netw Open.

[18] Doocy S, Lyles E, Roberton T, Akhu-Zaheya L, Oweis A, Burnham G (2015). Prevalence and care-seeking for chronic diseases among Syrian refugees in Jordan. BMC Public Health.

[19] Wilhelmsen L (1989). Risks of untreated hypertension. Discussion Hypertension.

[20] Nugent R, Barnabas RV, Golovaty I, Osetinsky B, Roberts DA, Bisson C, et al. Costs and cost-effectiveness of HIV/noncommunicable disease integration in Africa: from theory to practice. AIDS. 2018;32(Suppl 1):S83–92. 10.1097/QAD.0000000000001884 PMC6503960 29952794

[21] Njuguna B, Vorkoper S, Patel P, Reid MJA, Vedanthan R, Pfaff C, et al. Models of integration of HIV and noncommunicable disease care in sub-Saharan Africa: lessons learned and evidence gaps. AIDS. 2018;32(Suppl 1):S33–42. 10.1097/QAD.0000000000001887 PMC6779053 29952788

[22] Correia JC, Lachat S, Lagger G, Chappuis F, Golay A, Beran D, et al. Interventions targeting hypertension and diabetes mellitus at community and primary healthcare level in low- and middle-income countries:a scoping review. BMC Public Health. 2019;19(1):1542. 10.1186/s12889-019-7842-6 PMC6873661 31752801

[23] Augustovski F, Chaparro M, Palacios A, Shi L, Beratarrechea A, Irazola V (2018). Cost-effectiveness of a comprehensive approach for hypertension control in low-income settings in Argentina: trial-based analysis of the hypertension control program in Argentina. Value Health.

[24] Jafar TH, Islam M, Bux R, Poulter N, Hatcher J, Chaturvedi N (2011). Cost-effectiveness of community-based strategies for blood pressure control in a low-income developing country: findings from a cluster-randomized, factorial-controlled trial. Circulation..

[25] Seidman G, Atun R (2017). Does task shifting yield cost savings and improve efficiency for health systems? A systematic review of evidence from low-income and middle-income countries. Hum Resour Health.

[26] Mundeva H, Snyder J, Ngilangwa DP, Kaida A (2018). Ethics of task shifting in the health workforce: exploring the role of community health workers in HIV service delivery in low- and middle-income countries. BMC Med Ethics.

[27] Unger T, Borghi C, Charchar F, Khan NA, Poulter NR, Prabhakaran D (2020). 2020 International Society of Hypertension Global Hypertension Practice Guidelines. Hypertension..

[28] Prioreschi A, Munthali RJ, Soepnel L, Goldstein JA, Micklesfield LK, Aronoff DM (2017). Incidence and prevalence of type 2 diabetes mellitus with HIV infection in Africa: a systematic review and meta-analysis. BMJ Open.

[29] Nduka CU, Stranges S, Kimani PK, Sarki AM, Uthman OA (2017). Is there sufficient evidence for a causal association between antiretroviral therapy and diabetes in HIV-infected patients? A Meta-analysis. Diabetes Metab Res Rev.

[30] Hernandez-Romieu AC, Garg S, Rosenberg ES, Thompson-Paul AM, Skarbinski J (2017). Is diabetes prevalence higher among HIV-infected individuals compared with the general population? Evidence from MMP and NHANES 2009-2010. BMJ Open Diabetes Res Care.

[31] Tripathi A, Liese AD, Jerrell JM, Zhang J, Rizvi AA, Albrecht H, Duffus WA (2014). Incidence of diabetes mellitus in a population-based cohort of HIV-infected and non-HIV-infected persons: the impact of clinical and therapeutic factors over time. Diabet Med.

